# Clarification of the Dynamic Autothermal Thermophilic Aerobic Digestion Process Using Metagenomic Analysis

**DOI:** 10.1128/spectrum.00561-22

**Published:** 2022-03-29

**Authors:** Natsumi Ishida, Yoshihisa Kawano, Ryo Fukui, Min Zhang, Yukihiro Tashiro, Kenji Sakai

**Affiliations:** a Laboratory of Soil and Environmental Microbiology, Division of Systems Bioengineering, Department of Bioscience and Biotechnology, Faculty of Agriculture, Graduate School of Bioresources and Bioenvironmental Sciences, Kyushu University, Fukuoka, Japan; b Laboratory of Microbial Environmental Protection, Tropical Microbiology Unit, Center for International Education and Research of Agriculture, Faculty of Agriculture, Kyushu University, Fukuoka, Japan; University of Minnesota

**Keywords:** shotgun metagenomics, autothermal thermophilic aerobic digestion (ATAD), wastewater, antibacterial substance, nitrogen metabolism, organic acid degradation

## Abstract

This study details a unique process of autothermal thermophilic aerobic digestion (ATAD) of human excreta useful in producing nitrogen-rich and pathogen-free organic fertilizer. The process was divided into initial, middle, and final phases, based on changes in temperature, dissolved oxygen (DO), and bacterial community structure. The aim of this study was to determine bacterial factors that would affect liquid fertilizer production in the process, using shotgun metagenomic analysis of each phase. Although the abundances of all 28 gene categories include 4 categories in SEED subsystems level 1 were similar to those in another type of wastewater treatment system, the abundances of 4 gene categories changed remarkably. Among them, a decrease in the abundance of the phage-related gene category and the presence of antibacterial substances in secondary metabolism may explain the change in bacterial community structure from the material to the initial phase. Increases in the abundances of two gene categories, phage-related and secondary metabolism, coincided with a decrease in alpha diversity from the material to the initial phase. A potential increase in the abundance of genes in the category of sporulation from the middle to the final phase was correlated with deterioration of growth conditions and stabilization processes. In addition, prompt consumption of short-chain fatty acids in the initial phase and unusually stable ammonia accumulation throughout the process could be explained by the presence/absence of related metabolic genes. In conclusion, the relationships between bacterial function and unique characteristics of ATAD were revealed; our findings support the enhancement of liquid fertilizer production from wastewater.

**IMPORTANCE** Metagenome analysis was performed to determine the microbial dynamics of the unique autothermal thermophilic aerobic digestion process of human excreta, which includes initial, middle, and final phases. In this study, we revealed the details of functional genes related to physicochemical and bacterial characteristics in the ATAD process. Four gene categories showed increases and decreases during the digestion process. In addition, the unusual stable accumulation of ammonia and prompt consumption of short-chain fatty acids were explained by the absence or presence of related metabolic genes. In addition to revealing the relationships between bacteria and physicochemical properties, the results of this research may support improving wastewater management systems worldwide by using the ATAD process in liquid fertilizer production systems.

## INTRODUCTION

The United Nations General Assembly adopted sustainable development goals (SDGs) in 2015. This initiative consisted of 17 goals and 169 targets, including a universal call to action to end poverty, protect the planet, and ensure that all people enjoy peace and prosperity by 2030 (1). Specifically, goal 6 calls for clean water and sanitation, with a special focus on improving wastewater treatment. Wastewater results in environmental pollution without any treatment; therefore, to alleviate environmental deterioration and produce clean water, appropriate wastewater treatment processes are essential. Various aerobic and anaerobic microbial processes for wastewater treatment are known; they include anaerobic digestion (methane fermentation) (2), aerobic processes (activated sludge) (3), and autothermal thermophilic aerobic digestion (ATAD) (4).

ATAD has been used to produce liquid fertilizer from wastewater with high concentrations of organic matter. In this process, the temperature increases up to 55 to 70°C owing to mainly bacterial heat that oxidizes organic compounds without external heating, and pathogens and plant seeds are inactivated (5, 6). Compared with other wastewater treatment systems, there are several advantages of the ATAD process; for example, it has a simple controlling process with low facility costs, and it has a high degradation efficiency with a shorter treatment time (7). Digested wastewater following ATAD treatment can be directly utilized as a pathogen-free liquid fertilizer (8). The heat energy generated autothermally is comparable to the biogas energy obtained in aerobic digestion in a small-scale facility. Thus, this process may achieve zero wastewater discharge and can help accomplish goal 6 of the SDGs described above, even in developing countries.

Our previous research on a full-scale ATAD process in Chikujo Town, Japan, revealed a dynamic transition in bacterial community structure, with unique physicochemical properties, including temperature, dissolved oxygen (DO), oxidation-reduction potential (ORP), and ammonia nitrogen (Fig. S1 in the supplemental material) (9). Notably, previous research insisted that the changes of bacterial community structure were unique (10), and they were considered the result of the use of a self-inducing aerator (11). Based on the transitions in the bacterial community structure and several physicochemical parameters, we proposed three distinguishable phases: initial, middle, and final. To explain the changes in the bacterial community structure of the ATAD process, cell lysis activity assays and microcosm assays were performed (10). Moreover, activities against Gram-negative bacteria (but not Gram-positive bacteria) in the middle and final phases could be attributed to changes in the bacterial community structure. Another notable feature of the ATAD process is the stable accumulation of ammonia and rapid degradation of volatile fatty acids.

Although 16S rRNA gene amplicon analysis of bacterial community structure is a powerful tool for revealing a wide range of major and dominant bacteria in an open mixed-culture system (12, 13), their roles and functions can only be detailed indirectly from taxonomic results (14). To understand the functions of mixed cultures, gene-specific quantitative PCR (qPCR) and shotgun metagenomics have been employed in studies of methane fermentation (15), composting (16), and activated sludge (17). Gene-specific qPCR is considered a function-specific method because it can detect thoroughly annotated functional genes with low abundance, such as genes with functions in nitrogen metabolism (16) and antibiotic resistance (18). In contrast, shotgun metagenomics is a more comprehensive analytical method that theoretically covers all functional genes. Although the bacterial community structure in each phase was revealed using 16S rRNA gene amplicon analysis (9), bacterial functions that contribute to the formation of unique characteristics and changes in the bacterial community structure in the ATAD process have not yet been understood. Existing studies have prompted the search for bacterial functions in a transient mixed-culture system using shotgun metagenomics, and the results of such studies would help to improve manipulation of the ATAD process.

In this study, we applied shotgun metagenomics to determine the unique features of the ATAD system, with special attention to the mechanisms of changes in the bacterial community structure, stable ammonia accumulation, and rapid organic acid degradation.

## RESULTS

### Gene categories found in each phase.

**FIG 1:**
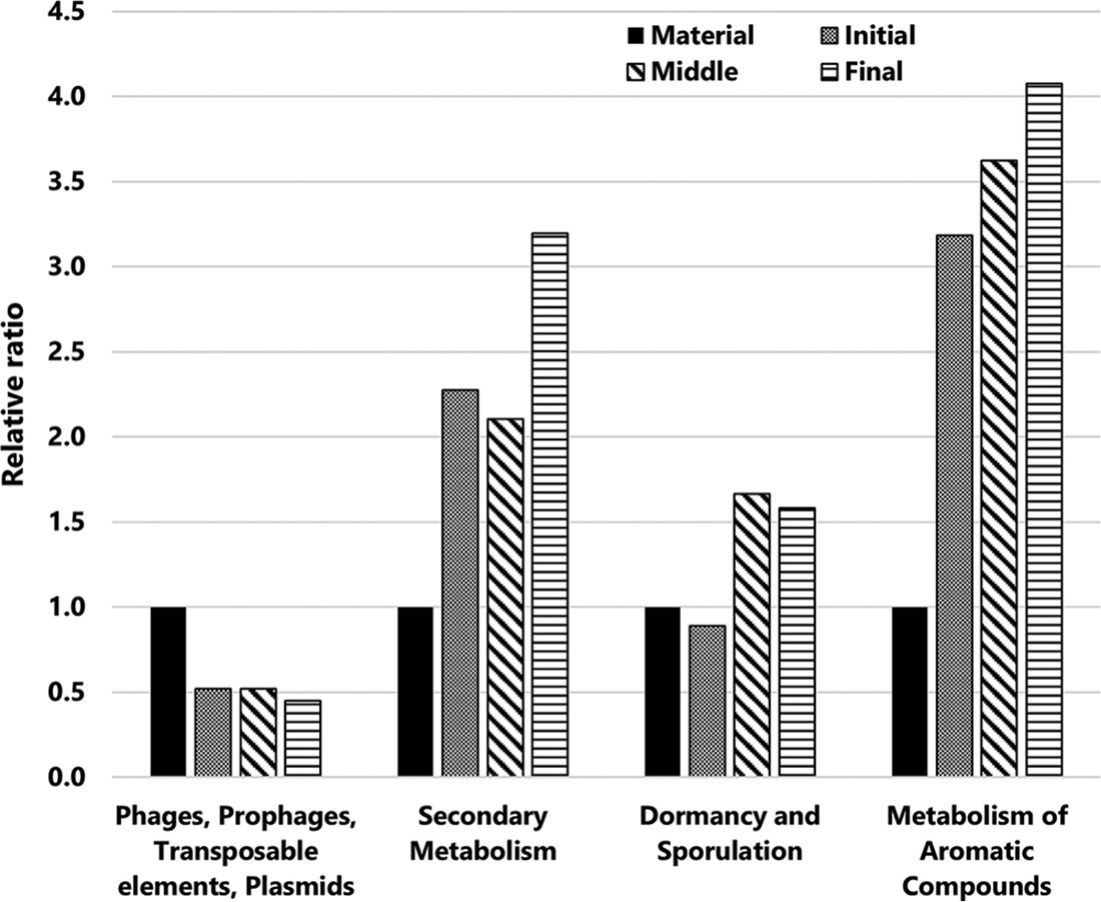
Classification of functional genes in each phase of the ATAD process and magnification of outstanding functional genes compared with those in the material. Bar graph patterns are detailed as follows: black bar, material; dotted bar, initial phase; diagonal line bar, middle phase; horizontal line bar, final phase.

Metagenome datasets of raw material (herein referred to as material) and ATAD samples in each phase were built using Illumina sequencing (Table S1). The assembly lengths calculated using Prokka Genome Annotation ranged from 44,192,569 to 76,612,235 bp. Although published guidelines for assessment of datasets obtained using shotgun metagenomics are lacking (19), rarefaction analysis of the annotated species richness indicated a saturation of each phase (Fig. S2). Therefore, we considered this dataset sufficient for metagenomic profiling and analysis of functional genes in each phase of the ATAD process. Figure S3 shows the abundance of all 28 annotated functional categories in SEED subsystems level 1, because this level was suitable for crude clarification of the transition. In these categories, amino acids and derivatives (9.0 to 9.8%), carbohydrates (10.8 to 11.9%), and protein metabolism (9.6 to 10.9%) were dominant in all phases. The abundances of these major categories were stable throughout the process. In contrast, Fig. 1 shows the predominant functional genes that changed from the material samples and indicates functional shifts in each phase. The four functional categories of (i) phages, prophages, transposable elements, and plasmids (phage related), (ii) secondary metabolism, (iii) dormancy and sporulation (sporulation related), and (iv) the metabolism of aromatic compounds (aromatic metabolism) showed increases or decreases in their abundance compared with those in the raw material (Fig. 1). For example, the relative ratio of phage-related genes decreased immediately in the initial phase, to less than half. In contrast, genes in the categories of secondary metabolism, sporulation, and aromatic metabolism increased by more than 1.5 times during digestion.

### Major functional genes in changed gene categories.

**FIG 2:**
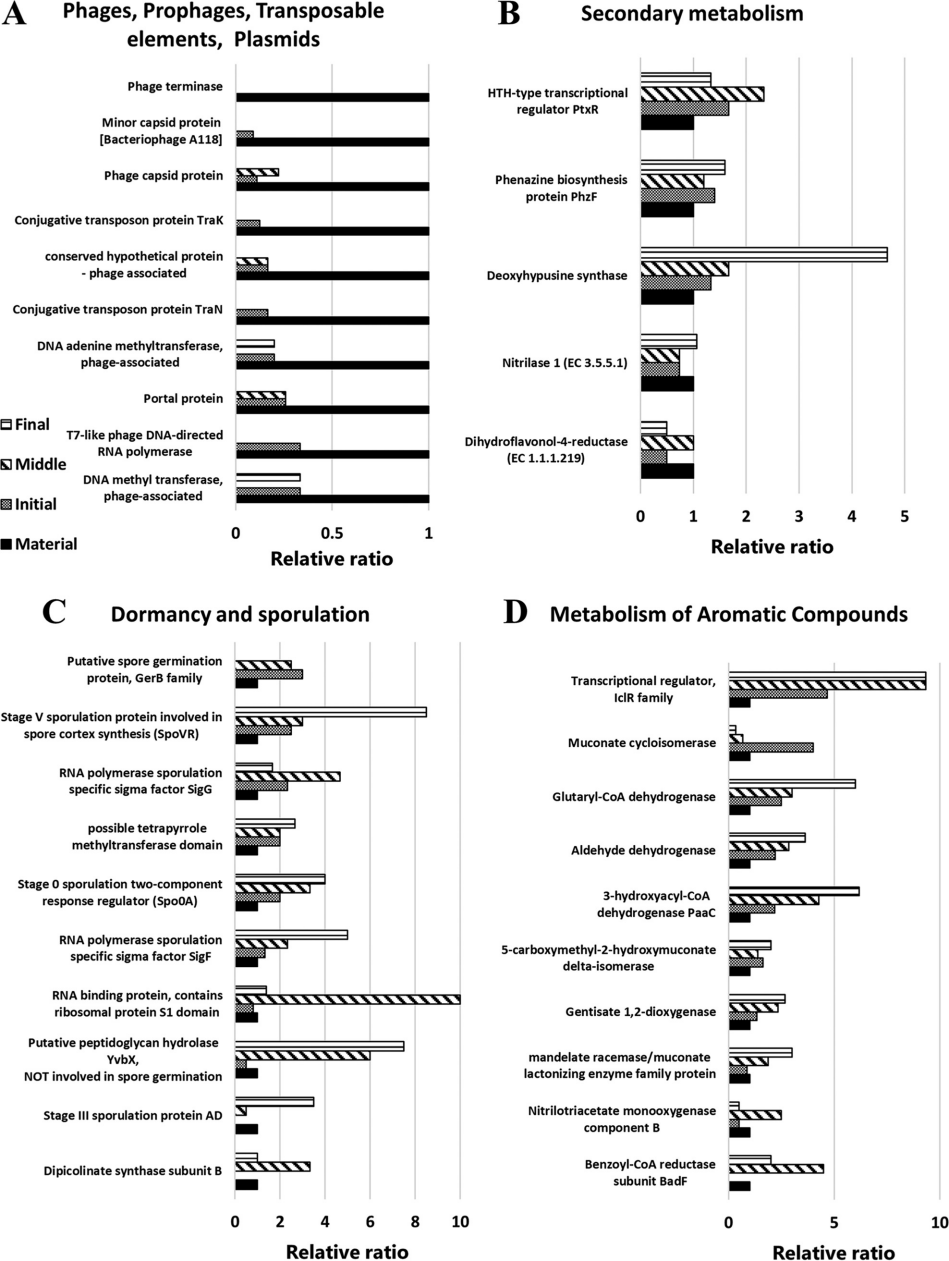
Predominant functions that changed from the material samples in SEED subsystems at function level of phage, prophage, transposable elements, plasmids (A), secondary metabolism (B), dormancy and sporulation (C), and metabolism of aromatic compounds (D). Bar plot patterns are the same as in Fig. 1.

Figure 2 shows the fluctuating functional genes in the four gene categories in SEED subsystems level 1 described in the previous section. The decrease in the phage-related gene category was caused by the reduction of almost all types of genes related to prophages, transposons, and plasmids (Fig. 2A). As described in more detail in Discussion, this means that a wide range of these mobile genetic elements are released at the initial phase, which is directly correlated with bacteriolysis. Genes in the secondary metabolism category also increased from the initial phase to the final phase. The predominant genes in secondary metabolism were related to paerucumarin biosynthesis (helix-turn-helix [HTH]-type transcriptional regulator PtxR), phenazine synthesis (phenazine biosynthesis protein PhzF), alkaloid biosynthesis (deoxyhypusine synthase), auxin biosynthesis (nitrilase 1), and tannin biosynthesis (dihydroflavonol-4-reductase) (Fig. 2B), and the enzymatic gene encoding deoxyhypusine synthase showed the most increased abundance in secondary metabolism. In this category, antibacterial substance production-related genes related to the biosynthesis of 10 antibiotics (phenazine, bacilysin, erythromycin, fosmidomycin, streptomycin, oleandomycin, puromycin, penicillin, tetracycline, and vancomycin) and three bacteriocins (linocin, colicin, and microcin) were found at each phase of the ATAD process. The antibacterial-related genes related to activity against Gram-positive bacteria that were detected with higher abundance were those for phenazine (abundances from material to final phase: 0.0047, 0.0073, 0.0065, and 0.0094%) and linocin (0.0031, 0.0051, 0.0014, and 0.0067%) (20, 21), whereas those against Gram-negative bacteria were erythromycin (0.0062, 0.0051, 0.0007, and 0.0013%), colicin (0.0016, 0.0073, 0.0036, and 0.0067%), and microcin (0.0109, 0.0029, 0.0058, and 0.0040%) (22, 23). Bacilycin, which has antibacterial activity against a broad range of bacteria, was also detected (0.0047, 0.0007, 0.0007, and 0%). Genes in the dormancy and sporulation categories decreased slightly from the material to the initial phase but increased from the initial to the middle phase. Almost all genes in this category were related to sporulation (Fig. 2C). In the aromatic metabolism category (Fig. 2D), the predominant genes that increased were mainly related to aromatic compound degradation. The categories of phage and secondary metabolism contributed to changes in bacterial community structure and unique physicochemical properties, whereas the categories of phage and sporulation were influenced by changes in the physicochemical properties of ATAD; more details can be found in Discussion.

### Genes for ammoniacal nitrogen metabolism.

**FIG 3:**
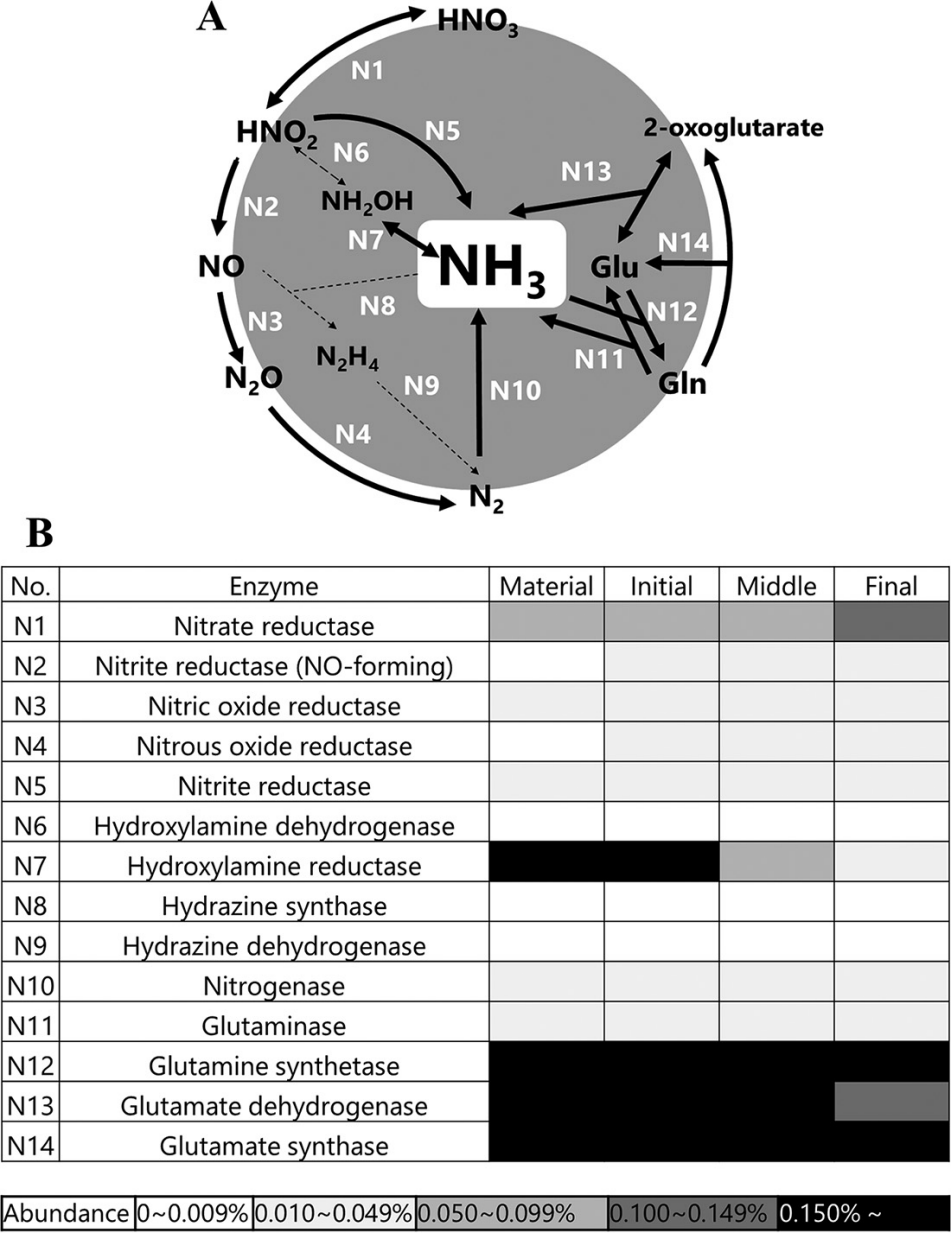
Nitrogen metabolic pathway. (A) Metabolic pathway. (B) Heat map of functions in nitrogen metabolism. Conversion pathway: denitrification (N1, N2, N3, N4), nitrification (N7, N6, N1), anammox (N8, N9). Production pathway: glutamate metabolism (N11-N14), nitrogen fixation (N10), nitrate reduction (N1, N5). The solid-line arrows indicate enzymatic genes that were detected. The small-dot line arrows indicate enzymatic genes that were not detected.

In our previous study, the concentration of ammoniacal nitrogen was maintained at a high level (ca. 1 g/L) throughout the ATAD process with slight fluctuations, and nitrite and nitrate ions were not detected at any time (Fig. S1G) (9). Thus, the gene category related to nitrogen metabolism was further analyzed to determine why the amount of ammoniacal nitrogen was stably maintained. Nitrogen metabolism mainly consists of nitrification, denitrification, anaerobic ammonium oxidation (anammox), nitrogen fixation, nitrate reduction, and glutamate metabolism. These processes are roughly classified into two groups for ammonia nitrogen metabolism: ammonia conversion (denitrification, nitrification, and anammox) and ammonia production (nitrogen fixation, nitrate reduction, and amino acid deamination, including glutamate metabolism). Figure 3A presents the ammonia metabolic pathway, and Fig. 3B shows the abundance of each enzymatic gene. In ammonia conversion, hydroxylamine dehydrogenase (N6), which is part of nitrification, was not observed in any phase (Fig. 3). The abundance of other nitrification enzyme genes was also not high (hydroxylamine reductase [N7], 0.05 to 0.16%, and nitrate reductase [N1], 0.08 to 0.12%). In addition, anammox enzyme genes, including those encoding hydrazine synthase (N8) and hydrazine dehydrogenase (N9), were not detected throughout the ATAD process. In contrast, some genes for denitrification enzymes (nitrate reductase [N1], nitrite reductase [NO forming] [N2], and nitric oxide reductase [N3]) were found in all phases. However, a gene related to nitrous oxide reductase (N4), which is also a key enzyme for denitrification, was observed to have low abundance (0 to 0.05%). These results support the idea that ammonia conversion would not work because of the absence or low abundance of the related pathways (N2, N4, N6, N8, and N9), which corresponded with the absence of nitrate (Fig. S1G).

In the ammonia production pathways, some enzyme genes related to glutamate metabolism, which is part of ammonification, were observed at a certain abundance throughout the process (glutaminase [N11], 0.02 to 0.03%, and glutamate dehydrogenase [N13], 0.13 to 0.18%). In contrast, the nitrogen fixation pathway was also observed (nitrogenase [N10], 0.01 to 0.03%), but nitrogen fixation would not occur owing to the high concentration of ammonia and aerobic conditions of the process (24). The nitrate reduction pathway also was observed in all phases (nitrite reductases [N1 and N5]); however, the substrate nitrate was not supplied owing to the lack of a nitrification pathway. These results suggest that the concentration of ammonia was maintained by glutamate metabolism, whereas the ammonia conversion pathways were not completely identified.

### Pathways of organic acid degradation.

**FIG 4:**
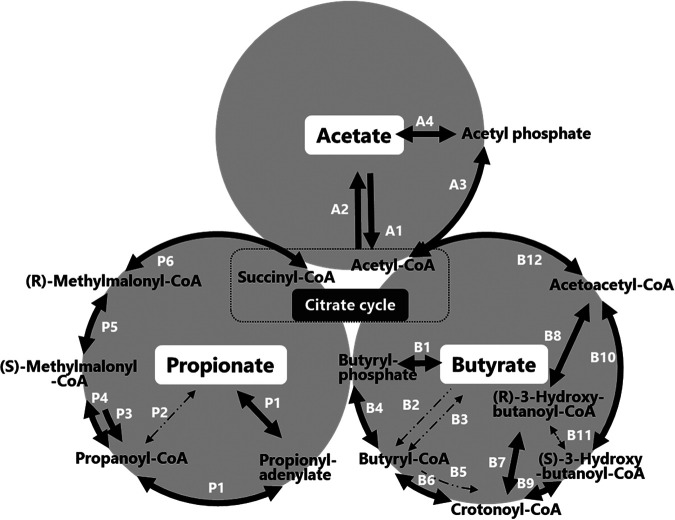
Metabolic pathways of organic acids in the initial phase. The solid-line arrows indicate enzymatic genes that were detected. The small, dash-dot-line arrows indicate enzymatic genes that were not detected.

A previous study showed that rapid consumption of organic acids, including acetate, propionate, and butyrate, occurred only during the initial phase, and no organic acids were detected thereafter (Fig. S1E) (9). Figure 4 shows the metabolic pathways of the respective organic acid degradations in the initial phase.

Acetate was mainly converted to acetyl-CoA synthetase (assigned number in Fig. S4, A1), and acetate kinase (A4) to phosphate acetyltransferase (A3) (Fig. 4). Genes for enzymes A1, A3, and A4 detected in the initial phase were found (Fig. S4). As shown in Fig. 4, propionate degradation is mainly initiated by acetyl-CoA synthetase (P1) or propionate CoA transferase (P2), connecting to P3 to P6. The results of shotgun metagenomics indicated complete degradation pathways. The butyrate degradation pathway also consisted of multiple pathways, initiating with butyrate kinase (B1) and ending with acetyl-CoA, as shown in Fig. 4. The results of shotgun metagenomics showed a complete degradation pathway ([B1, B4, B6-1, B7, B8, and B12] and [B1, B4, B6-1, B9, B10, and B12]). Thus, shotgun metagenomics revealed complete degradation pathways of major organic acids.

## DISCUSSION

In a mixed-culture system, various types of interactions occur among bacteria, and physicochemical changes affect the bacterial community and vice versa. The potentially existing total functions of the mixed-culture system can be estimated from the total metagenomics of the system. The abundances of functional genes reflect all aspects of the system bidirectionally, and changes in the abundances of functional gene groups, as well as of members of the bacterial community, would reflect the events that occurred in the process. In this study, we performed shotgun metagenomic analyses of each phase of a unique ATAD process (9) to determine the reasons why the physicochemical properties of the process are unique following changes in the bacterial community. The ATAD process stably maintained a high concentration of ammonia, rapidly consumed organic acid, and drastically transitioned the bacterial community structure, constituting not-yet cultured members (9). In this section, we discuss each point with a focus on changes in functional gene components, which may reflect physicochemical properties and the bacterial community structure in the ATAD process.

We compared the bacterial community structure as obtained via shotgun metagenomics and 16S rRNA gene amplicon analysis. Although we used identical samples for each experiment, the bacterial community structure was considerably different at the phylum level (Fig. S5). Studies using two next-generation sequencing techniques have reported that these differences were observed in other samples (freshwater and chicken gut), and some variables (database of strains, data size, PCR biases, and horizontal gene transfer) can affect the results for bacterial community structure (25, 26). We employed EzBioCloud (registered genera, 11,446) (27) for 16S rRNA gene amplicon analysis and RefSeq (registered genera, 2,293) (28) for shotgun metagenomics. Therefore, significant differences in the registered information in the database would result in mismatches in the bacterial community structure between the two sequencing methodologies, even at the phylum level. Furthermore, because the changes in bacterial community structure between RefSeq and 16S rRNA sequence obtained in the shotgun metagenomics dataset were similar (Fig. S5 and S6), PCR biases would also cause the discrepancy between 16S rRNA gene analysis by shotgun metagenomics and 16S rRNA gene amplicon analysis. In fact, using shotgun metagenomics (data not shown), several obligate anaerobic clades, such as the family *Clostridiaceae*, were found to be abundant in the middle and final phases, when the DO values (Fig. S1B) indicated aerobic conditions, whereas these obligate anaerobic clades were not abundant in the middle and final phases using 16S rRNA gene amplicon analysis (9). Therefore, the mismatch would result in misinterpretations that the genes derived from facultative anaerobic or aerobic clades were affiliated with obligate anaerobic clades. This mismatch problem can be solved by growing the database.

Compared with the gene categories in each phase, some gene categories consistently showed high abundance throughout the ATAD process (Fig. S3). Similar results were also reported in methane fermentation (29, 30) and activated sludge processes (31), which confirmed that those consistently high-abundance categories were biogenic factors without a dependence on the physicochemical characteristics and the bacterial community structure of the process. During the ATAD process, four categories showed fluctuations in their abundances (Fig. 1 and 2). Changes in the categories of aromatic and secondary metabolism have been reported in methane fermentation (29) and activated sludge (31). High temperatures affect prophage induction in Escherichia coli (32), and mobile genetic elements in daily manure are degraded by thermophilic anaerobic digestion (33). Decreases in the abundances of phage-related genes in the ATAD process would reflect prophage induction and bacteriolysis in host cells by phages or prophages and degradation of those gene-encoded factors under thermophilic conditions. Moreover, previous research has shown fluctuations in the numbers of viable cells in the ATAD process (9). Because the occupancy of prophage and the numbers of prophage genome elements vary for each bacterium (34), we could also hypothesize that the number of bacteria encoding the prophage gene has decreased. Deoxyhypusine synthase-encoding gene in secondary metabolism was mainly found in archaea in the ATAD process (Fig. 2B). Hypusine is an unusual amino acid produced by this enzyme and deoxyhypusine hydroxylase (35). Those enzymes are conserved in eukaryotic initiation factor 5A (eIF5A), which is related to cell growth and viability; however, the behavior of this protein is not well understood in archaea (36). In contrast, genes related to the biosynthesis of antibiotics (phenazine) were abundant in the secondary metabolism category. Phenazines also act as intercellular signals and substrates for intercellular redox transformations (37); therefore, this substance not only has antibacterial activity but also facilitates redox homeostasis. Various biosynthesis genes related to antibiotics and bacteriocins were detected in each phase. Our previous report indicated lytic activity toward only Gram-negative bacteria in the middle and final phases (10). Colicin, microcin, and erythromycin, which have shown antibacterial activity against Gram-negative bacteria, and biosynthesis genes of those antibiotics, were detected in the middle and final phases. Tian et al. reported that antibiotics are a key factor in the changes in bacterial community structure in an activated sludge process (18). Consequently, we hypothesized that the emergence of prophage and biosynthesis of antibacterial substance would induce antibiotic activity against bacteria, which would result in transitions of the bacterial community structure in the ATAD process.

Another gene category of sporulation that had increased abundances was generally related to spore formation (Fig. 2C), which is induced in response to unfavorable environmental conditions, such as nutrient limitation and changes in temperature and pH (38). Therefore, the abundances of the genes in the ATAD process possibly increased owing to successive changes in or deterioration of the growth environment, such as increasing temperature (Fig. S1A), rising pH (Fig. S1C), and decreases in carbon sources (Fig. S1D). Sporulation genes were increased in a mixed culture as a stress response to thermophilic conditions (39). The observation that changes in both physicochemical properties and bacterial community structure occur until the end of the initial phase is supported by the increase in sporulation-related genes.

Next, we discuss the gene categories of nitrogen metabolism in the ATAD process. The concentration of ammonia was stably accumulated, and nitrate was not found throughout the ATAD process (9). Although the bacterial community structure changed drastically from the initial to the final phase, the chemical form of the nitrogen compounds did not change, with slight fluctuations (Fig. S1G). From the metagenomic structure, we inferred that genes related to glutamate metabolism were higher in abundance (Fig. 3). This suggests that ammonia could possibly be produced by glutamate metabolism originating from organic nitrogen. However, owing to the absence of hydroxylamine dehydrogenase (N6) (Fig. 3), the nitrification pathway was not completed in any phase. In a previous study, no nitrification bacteria were found, with accumulations of scarce amounts of nitrate throughout the ATAD process, even under aerobic conditions (9). Denitrification enzyme-coding genes were found in the initial and middle phases, even though their abundance was low (Fig. 3B). Therefore, the lack of a nitrification pathway is the main reason for the absence of nitrate in the process, which is distinguishable from another wastewater treatment system in which nitrification pathways were found (16, 40). The stable and high concentrations of ammonia obtained through the ATAD process would be stabilized via ammonia assimilation and glutamate metabolism, with a smaller contribution from nitrification.

Another specific feature of ATAD is the prompt consumption of organic acids in the initial phase (9). The degradation process of organic acids was detailed in another wastewater treatment system (41, 42); however, their metabolism in the ATAD process was not revealed. Shotgun metagenomics detected complete degradation pathways of the three volatile fatty acids (Fig. S4).

**FIG 5:**
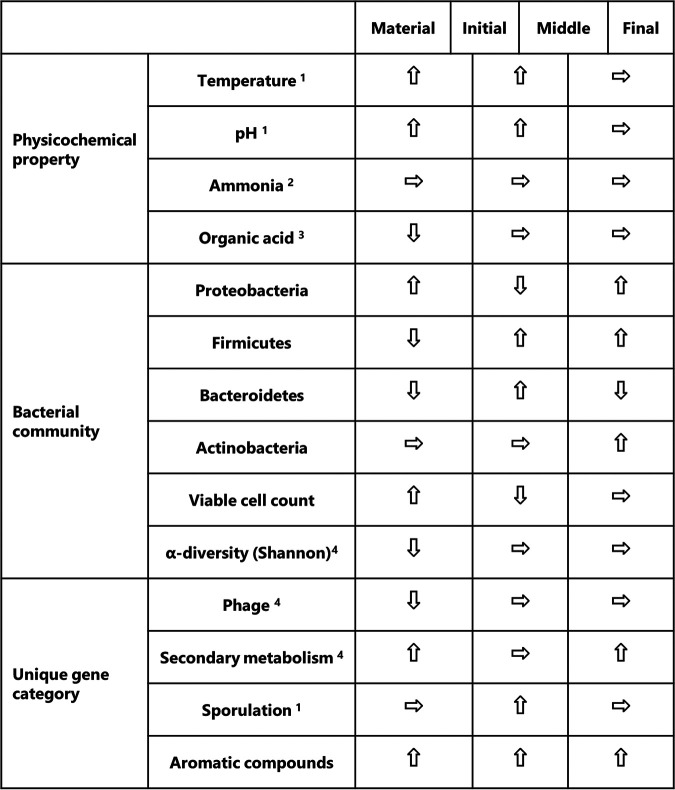
Comprehensive perspective of three factors in the ATAD process. The arrow direction indicates the shift of each factor between the previous phase and the following phase. Data for physicochemical property and bacterial community are from Tashiro et al. (9). Numerical superscripts denote the relation between a physicochemical property or a bacterial community and functional genes, as follows: 1, relation of sporulation with changes of temperature and pH; 2, relation of glutamate metabolism with stable ammoniacal nitrogen; 3, relation of organic degradation pathways with rapid consumption of acetate, propionate, and butyrate; 4, relation of prophage emergence and biosynthesis of antibacterial substances with transitions of the bacterial community. Four phyla in the bacterial community changed in abundance.

In conclusion, we demonstrated the complete time-series of causal relationships of phenomena occurring in the unique ATAD process with changes in the metagenomic structure, as summarized in Fig. 5. Relationships between each element of three factors, i.e., changes in the bacterial community structure, stable ammonia accumulation, and organic acid consumption, are mutually connected and are almost comprehensive. By commencing self-inducing aeration with circulation, rapid growth of the Gram-negative bacterial group was initiated through consuming volatile fatty acids. This led to increases in temperature and pH and a decrease in DO, which resulted in the release of mobile genetic elements that accompanied cell lysis and the growth of another thermotolerant bacterial group. During the process, ammonia was provided from organic nitrogen compounds and used as a nitrogen source repeatedly, which resulted in a superficially stable accumulation of ammonia throughout the process. We recently reported that the self-inducing aerator, a Venturi-type aeration and mixing device, is the key element in reproducing the unique ATAD process (11). Various kinetic studies on microbial structure, metagenomic analysis, and metatranscriptomes are needed to further understand the bioprocess and actual function in a mixed-culture system.

## MATERIALS AND METHODS

### Sample collection.

Samples were collected from the full-scale ATAD facility of human excreta sludge equipped with a self-inducing aerator, which is located in Chikujo Town, Fukuoka Prefecture, Japan. We used the same samples in this study as in the previously reported research, and the operating conditions of the ATAD process have been described previously (9). Briefly, collected human excreta samples were digested by mixing and aeration using an aerator instrument, and this procedure was performed for approximately 3 weeks. The ATAD samples from the material, initial, middle, and final phases were collected on days 0, 1, 3, and 9 from 17 to 26 June 2014. These samples were stored at −20°C until DNA extraction.

### DNA extraction and library preparation.

DNA was extracted from ATAD samples using the DNeasy PowerSoil kit (Qiagen, Hilden, Germany) according to the manufacturer’s instructions. The concentration of extracted DNA was measured using a Qubit Fluorometer (Thermo Fisher Scientific, MA, USA) with a Qubit dsDNA HS assay kit (Thermo Fisher Scientific). DNA libraries for shotgun metagenomics were prepared using the Nextera XT DNA Library Prep kit (Illumina, Inc., CA, USA), and DNA libraries were amplified using a TaKaRa PCR Thermal Cycler Dice Gradient (TaKaRa Bio, Inc., Shiga, Japan) and utilizing the Nextera XT DNA Index kit (Illumina, Inc.) to attach index sequences that distinguished the samples. PCR products were purified using Agencourt AMPure XP (Beckman Coulter, Inc., CA, USA). To measure the DNA concentrations of the prepared libraries, qPCR was performed using the CFX Connect Real-Time System (Bio-Rad Laboratories, Inc., CA, USA), a GeneNext NGS Library Quantification Kit (Toyobo Co., Ltd., Osaka, Japan), and KOD SYBR qPCR mix (Toyobo Co., Ltd.).

### Shotgun metagenomics.

The prepared DNA libraries were mixed with the MiSeq reagent kit v3 (Illumina, Inc.) for sequencing. The DNA libraries were sequenced using Illumina MiSeq (Illumina, Inc.). Approximately 1 to 2 Gb of sequencing data was obtained for each ATAD sample. The raw sequence data were processed using FASTQ Toolkit (version 2.2.0) for adapter trimming, quality, trimming, and length filtering. The processed dataset was applied to the SPAdes Genome Assembler (version 3.9.0) to assemble small genomes from bacterial datasets, and the assembly length was calculated via Prokka genome annotation to identify coding sequences in genomes. Those three software programs were obtained from Illumina BaseSpace. Reads assembled by SPAdes Genome Assembler were applied to MG-RAST (https://www.mg-rast.org/) (43) and annotated using SEED subsystems in MG-RAST for functional analysis; functional genes were annotated when their similarity was over 60%. The corresponding taxonomic information for the assembled reads was annotated using RefSeq in MG-RAST, and rRNA sequence data from shotgun metagenomics was applied to EzBioCloud (27) for taxonomic analysis. The bacterial community structure obtained by RefSeq and rRNA data from shotgun metagenomics was compared with the result of 16S rRNA gene amplicon analysis. Using MG-RAST, the rarefaction curve was calculated based on annotated species richness. BLASTn (https://blast.ncbi.nlm.nih.gov/Blast.cgi) was used to identify specific functional genes. KEGG Pathway Database (https://www.genome.jp/kegg/pathway.html) was used to draw the metabolic pathways of nitrogen and organic acids. UniProt (https://www.uniprot.org/) was used as a reference for protein functions. The ratio of total annotated reads was calculated from SEED subsystems level 1 data using the following formula: ratio of total annotated reads (%) = number of representative hits for specific gene category/number of representative hits for all gene categories × 100.

### Data availability.

Raw read sequences obtained via Illumina MiSeq were deposited under BioProject identifier (ID) PRJDB12439 in the DNA Data Bank of Japan, and the DRA accession number is DRA012923.
