# Supplementary material for: Clarification of the Dynamic Autothermal Thermophilic Aerobic Digestion Process Using Metagenomic Analysis
Source: Microbiol Spectr. 2022 Mar 29;10(2):e00561-22. doi: 10.1128/spectrum.00561-22 (PMC9045309; doi:10.1128/spectrum.00561-22)
Supplement: SUPPLEMENTAL FILE 1 — Supplemental material. Download SPECTRUM00561-22_Supp_1_seq5.pdf, PDF file, 0.6 MB [file spectrum00561-22_supp_1_seq5.pdf]

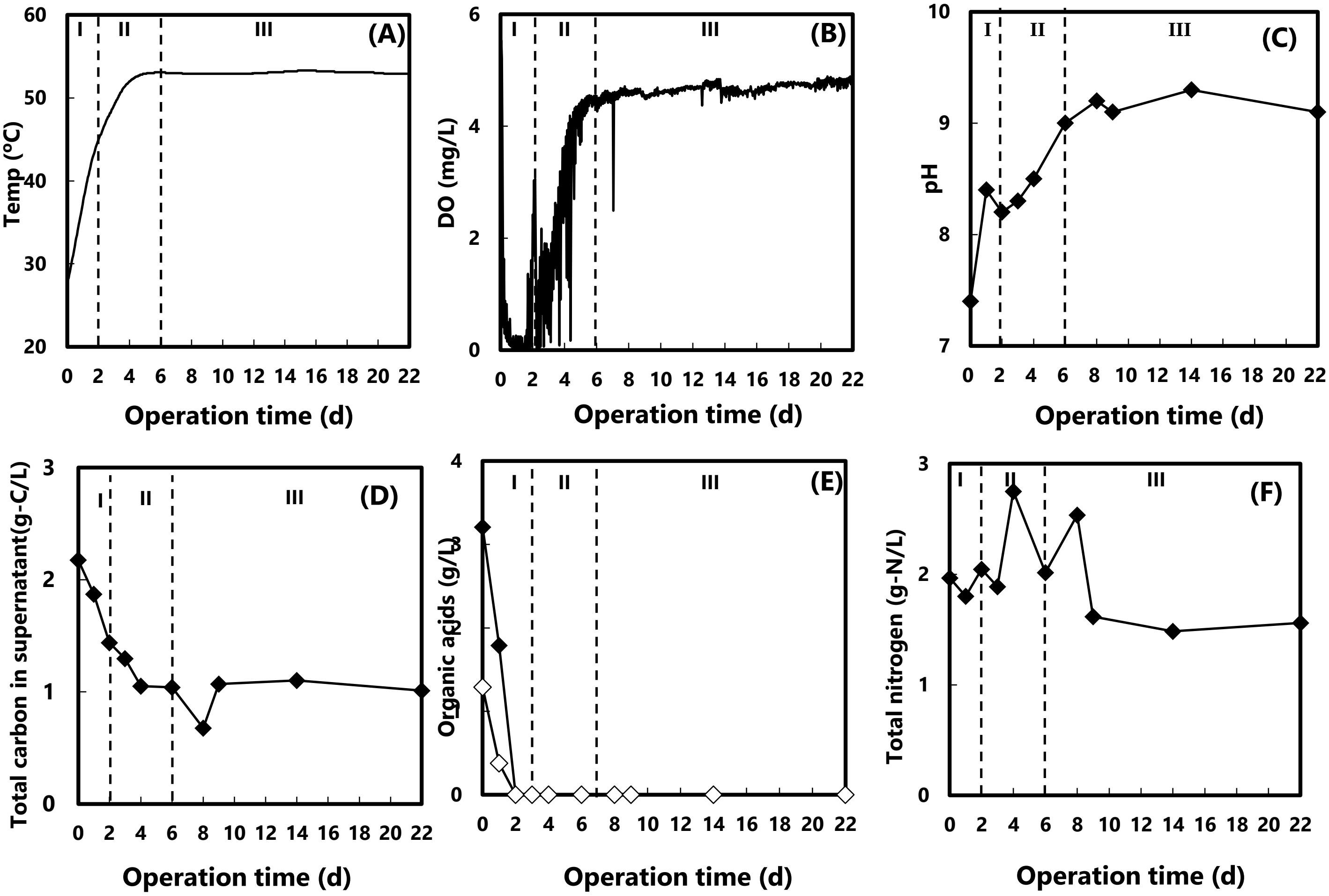

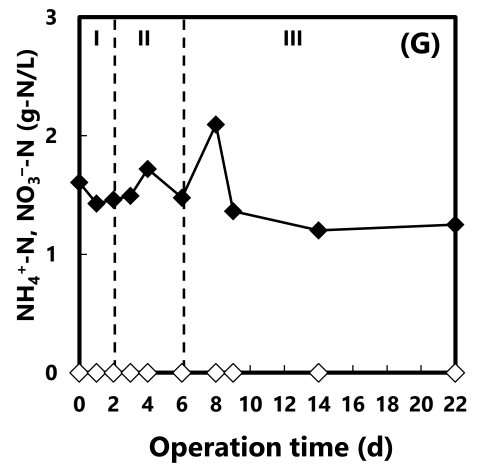

Fig. S1 Change of physicochemical parameters in ATAD process of human excreta in 2014 (cited from Tashiro et al., 2018). (a) temperature; (b) DO; (c) pH; (d) total carbon in supernatant; (e) organic acids in supernatant; (f) total nitrogen; (g) ammoniacal nitrogen and nitric acid nitrogen in supernatant; Symbols in (e): closed rhombus, acetic acid; open rhombus, propionic acid. Symbols in (g): closed rhombus, ammonia; open rhombus, nitric acid nitrogen. I: initial phase, II: middle phase, III: final phase.

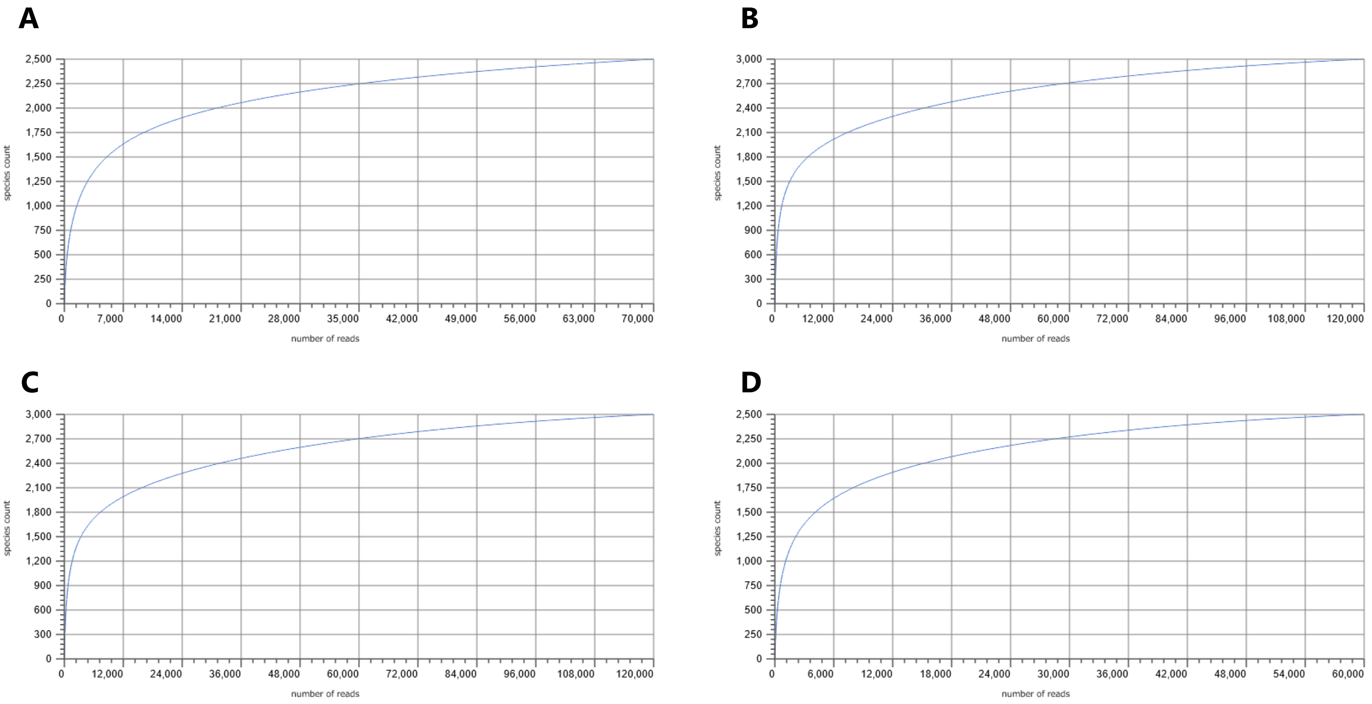

Fig. S2 The rarefaction curves of annotated species richness on MG-RAST. (A) material, (B) initial phase, (C) middle phase, (D) final phase.

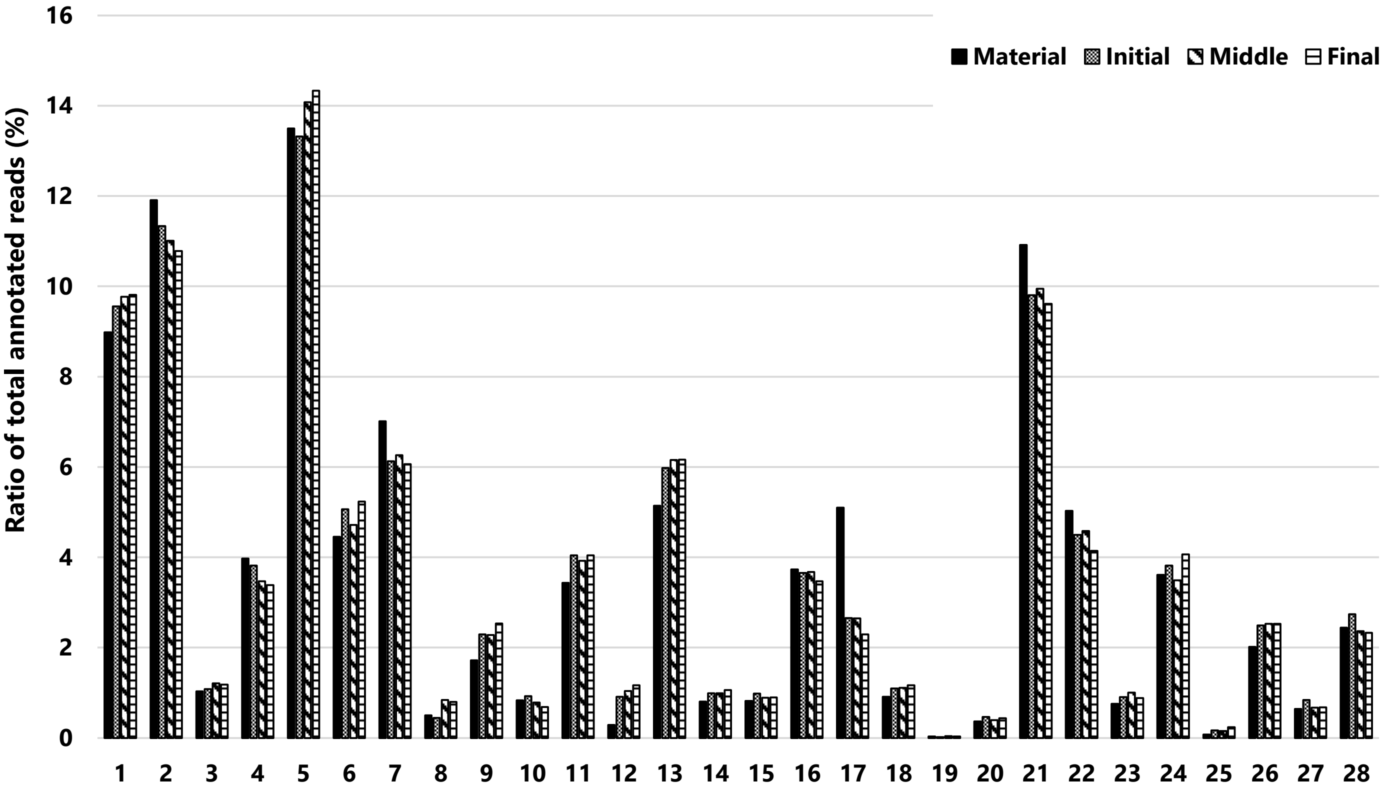

Fig. S3 Classification and ratio of functional genes in each phase of the ATAD process on SEED subsystems level 1.

1: Amino acid and derivatives, 2: Carbohydrates, 3: Cell division and cell cycle, 4: Cell wall and capsule, 5: Clustering based subsystems, 6: Cofactors, vitamins, prosthetic groups, and pigments, 7: DNA metabolism, 8: Dormancy and sporulation, 9: Fatty acids, lipids, and isoprenoids, 10: Iron acquisition and metabolism, 11: Membrane transport, 12: Metabolism of aromatic compounds, 13: Miscellaneous, 14: Motility and chemotaxis, 15: Nitrogen metabolism, 16: Nucleosides and nucleotides, 17: Phage, prophage , transposable elements, plasmid, 18: Phosphorus metabolism, 19: Photosynthesis, 20: Potassium metabolism, 21: Protein metabolism, 22: RNA metabolism, 23: Regulation and cell signaling, 24: Respiration, 25: Secondary metabolism, 26: Stress response, 27: Sulfur metabolism, and 28: Virulence, disease and defense.

Bar graph patterns are detailed as follows: Closed bar: material, polka dot bar: initial phase, diagonal line bar: middle phase, horizontal line bar: final phase.

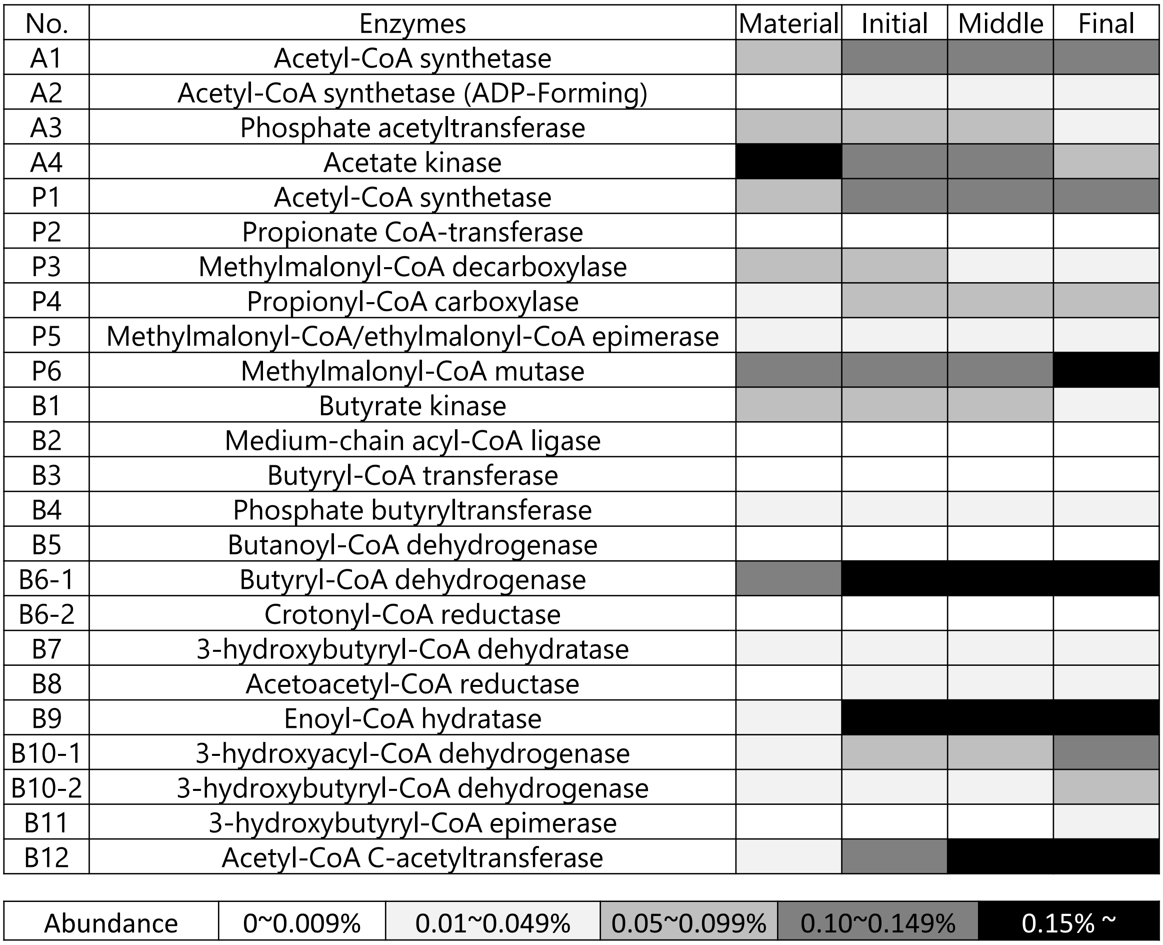

Fig. S4 Heat map of enzymes which belong to organic acid degradation pathways. Bottom column indicate color chart of heat map.

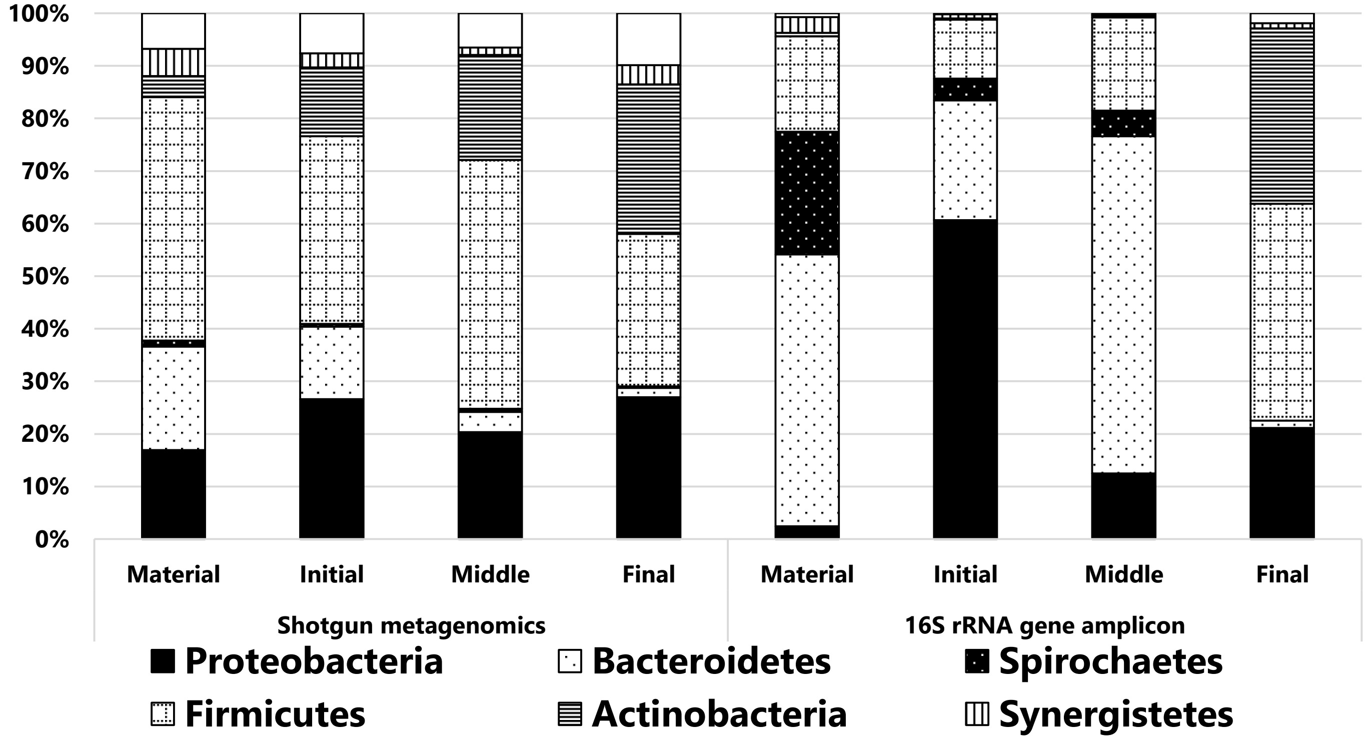

Fig. S5 Comparison of change in bacterial community structure during the ATAD process at the phylum level.

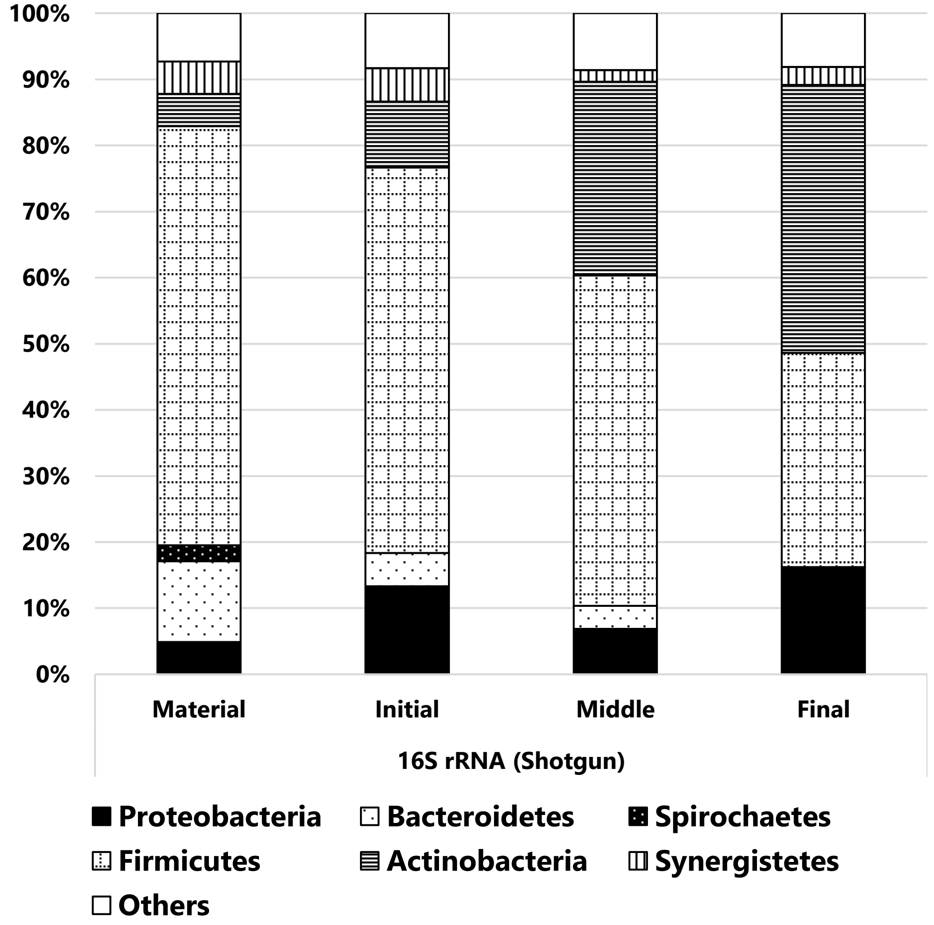

Fig. S6 Change in bacterial community structure during the ATAD process at the phylum level using 16S rRNA sequencing from shotgun metagenomics data.

Table S1 General information of shotgun metagenomics from ATAD samples (Prokka annotation, MG-RAST)

Phase	Prokka annotation				MG-RAST	
					Predicted protein	
	CDS	Assembly length	Contigs	rRNA	Known function	Unknown function
Material	41739	44192569	65540	134	38313	27140
Initial	69834	76612235	100666	281	77717	22757
Middle	70855	74199882	102031	307	77353	24447
Final	49262	50541108	52835	176	42644	10050
